# Notes and News

**Published:** 1898-04-30

**Authors:** 


### NOTES AND NEWS.

(Collected and Communicated.)

H.R.H. the Duchess of Fife will open the bazaar at the Empress Rooms on May 4th in aid of the Homes for Waifs and Strays. Two thousand three hundred children are cared for by this charity.

At the Festival Dinner of the East London Hospital for Children, Viscount Falkland suggested the erection of a whooping-cough ward, in view of the excessive mortality of this disease among the children in the East-end.

The new "Agnes Barr" Dispensary, in connection with the Glasgow New Samaritan Hospital, will be opened in May. The new buildings connected with this hospital include a model steam laundry, and many other modern equipments and improvements.

A challenge-cup has recently been presented by the Great Northern Railway Company's chief officials, to be competed for annually by the ambulance men in the employ of the company. After many competitions, the King's Cross team has been adjudged the winner.

At a recent inquest Dr. Danford Thomas stated that in London between 500 and 600 infants were suffocated in bed, while in the United Kingdom there were some 1,500 annually. Among the upper classes such deaths were unknown.

A protest has been addressed to the Home Secretary by the Committee of the London Anti-Vivisection Society against his action in registering the British Institute of Preventive Medicine on the Thames Embankment as a place at which experiments on animals may be performed. After most careful inquiry, the Home Secretary has found no reason to alter his decision.

The Duke and Duchess of Marlborough are to pay a visit on May 12th to the Colony for the Employment of Epileptics at Chalfont St. Peter. The Duke will lay the memorial stone of a home for epileptic boys, and the Duchess is to open the Victoria House, one of the homes for epileptic men. Mrs. Passmore Edwards, on the same date, will lay the foundation-stone of a new home for epileptic girls.

The cases of plague at Jiddah are decreasing in a most satisfactory manner.

At the eighty-fourth festival dinner of the Royal Hospital for Diseases of the Chest, City Road, subscriptions amounting to £4,400 were announced.

The thirtieth annual banquet in aid of the French Hospital and Dispensary takes place at the Hotel Cecil on May 7th.

The Countess Murphy and a few friends have subscribed upwards of £2,000 for building a chapel and a mortuary for the South Infirmary, Cork.

Mr. Chisholm Williams will lecture on "Angular Curvature of the Spine," at the City Orthopædic Hospital, on Tuesday, May 3rd, at half-past five p.m.

The teaching of ambulance work has been carried to a high pitch of efficiency in the Spanish army, and every soldier is well qualified to render very effectual first aid to wounded soldiers.

H.R.H. Princess Henry of Battenberg has sent a donation to the "Mother's Cot" Fund of the Children's Ward, Royal Isle of Wight Infirmary and County Hospital.

The Metropolitan Asylums Board has decided to build an ophthalmic hospital school at Brentwood, in small cottages, each cottage to contain about ten children, and the whole set of cottages to be limited to 270 inmates.

An appeal is being made for a sum of £2,000 to cover the cost of an additional ward, nurses' quarters, a new operating theatre, chapel, and mortuary for the St. Mary's Hospital for Sick Children, Plaistow. It is found that the hospital, as it stands, does not nearly meet the demands on its beds, and it is hoped the money will be forthcoming to allow of the necessary enlargement.

The Prince of Wales opened the Royal Photographic Society's International Exhibition at the Crystal Palace on the 25th inst., when, making a tour of inspection of the exhibition, the Prince visited the exhibit of the Prince of Wales's Hospital Fund. Amongst the photographs shown of those who have joined the Great Roll of Ministering Children were portraits of all the Royal children. The Prince was much pleased to see a practical demonstration of the interest taken in the movement by the children of England. When the roll contains 100,000 names an album, containing the portraits of all the young subscribers to the hospitals, will be presented to him.

Mr. Alexander Peckover, the Lord Lieutenant of Cambridgeshire, has made a most generous gift of £4.000 to the Eastern Counties Asylum for Idiots and Imbeciles, the sum to be expended in the technical training of those imbeciles who are capable of instruction in carpentry, basket-weaving, wood-carving, and similar industries. This institution is admirably managed, and has for some years set an excellent example of what may be done in the direction of manual training for the mentally deficient. There are large possibilities in such training; but, unfortunately, in many asylums very little is done in this direction. At Earlswood, the standard of technical arts is exceptionally good, and everything that patience and perseverance can suggest is done to encourage the patients to develop any small talent which they may possess.

